# Awareness of Health Insurance among Inpatients at a Tertiary Care Hospital in Coastal Karnataka

**DOI:** 10.4103/0970-0218.69288

**Published:** 2010-07

**Authors:** B Reshmi, Ragil Raghunath, B Unnikrishnan

**Affiliations:** Department of Health Information Management, Manipal College of Allied Health Sciences, Manipal, Karnataka, India. E-mail: reshmi.b@manipal.edu; 1 Department of Community Medicine, Kasturba Medical College, Mangalore, Manipal University, India

Sir,

The rise in health care demand has increased the cost of health care system to the extent that specialized care is beyond the reach of common man, only 10% of the Indians have some form of health insurance, mostly inadequate.(1)With this background, the present study was undertaken so that, patients coming to a hospital for treatment would be an appropriate target group to assess awareness toward health insurance.

This study was carried out at Kasturba Hospital, Manipal, Karnataka – a 1470 bedded teaching and referral hospital. The study subjects included 340 patients admitted in the hospital, selected by simple random sampling method from the list of inpatients in the hospital. Data were collected with the help of a pre-tested semi-structured questionnaire.

The data were analyzed using SPSS version 11.5. Socio-economic status of the respondents were assessed using modified Kuppuswamy urban scale.(2)Chi square test for association was carried out to find out the association between socio-economic status and awareness of health insurance.

The males constituted 72.1% and females constituted 27.1% of the total respondents. Majority were in the age group of 20-29 (30.8%), respondents belonging to the lower class were more (52.4%) compared to other socio-economic groups.

Table 1depicts the awareness of health insurance. It was found that 62% of the respondents were not aware of health insurance. Among those who were aware of health insurance, about 34.1% of the respondents said that media was the source of information, followed by insurance company (31.1%) and peers and relatives (28.5%).

**Table 1:** Awareness of health insurance *vs* socio-economic status of the respondents

Socio-economic status	Awareness of health insurance (*N*=340)		
	Aware No (%)	Not aware	Total
Upper	16 (64)	9 (36)	25
Upper middle	62 (45.6)	75 (55)	137
Lower middle	21 (24.4)	65 (75.6)	86
Upper lower	31 (33.7)	61 (66.3)	92

Chi-square - 21.846 *P*=0.0001

The respondents belonging to the upper (64%) and middle (70%) socio-economic groups were more aware as compared to the lower group (33.7%) which was found to be statistically significant.

A study on awareness about health insurance carried out at Jaipur city of Rajasthan state in India shows 43.4% were aware of health insurance,(3)another study on the awareness and attitude of the general public toward health insurance among people of Mangalore city shows 64% of the respondents were aware of health insurance.(4)In the present study, the awareness was found to be low probably because of the fact that about 53% of the population were from the lower socio-economic status. Media seemed to have played an important role in dissemination of information. A study conducted in Gujarat(5)found out that the need for education for rural and urban population alike on the concept of health information is a crucial aspect on extending awareness about health insurance on a large-scale. This calls for effective information, education and communication activities which will improve understanding of insurance by the public and hence awareness of health insurance will also improve.

The findings from the present study will be an eye opener to know where the patients stand with regard to their knowledge about health insurance covering the medical expenses. It can also help the hospital administration to become aware of the present status of health insurance awareness among the patients and take the necessary steps to make them aware of the need for health insurance to meet the ever rising medical expenses in view of unpredictable illness and injuries to which anyone can be a victim.
